# Image based quantification method reveals differential patterns of lip desquamation associated with age and sex

**DOI:** 10.1038/s41598-025-89264-x

**Published:** 2025-04-08

**Authors:** Hanji Kim, Jung Yeon Seo, Sangseob Leem, Seung Won You, Yunkwan Kim, Nae Gyu Kang

**Affiliations:** Research and Innovation Center, R&D Institute, LG Household & Health Care (LG H&H), Ltd, Seoul, 07795 Republic of Korea

**Keywords:** Image processing, Skin manifestations, Biological techniques

## Abstract

The rough surface of lip skin is a dermatological and aesthetic issue that affects facial appearance. While several studies have addressed this common problem, none of them have exclusively utilized image analysis techniques. In this study, we aimed to quantify lip desquamation from facial images in a total of 55 participants and compare the results to those obtained from the tape-stripping method. The central region of the lower lip was extracted from each facial image, and global thresholding was applied using the threshold value T, which is defined as Mean + 2.5 SD. In this equation, Mean and SD represent the average and standard deviation of each grayscale values within the image, respectively. The desquamation rate was calculated as the proportion of pixels corresponding to desquamated flakes within the analyzed region. We found that our approach accurately represented the lip surface states, with a strong correlation with visual assessment (VA) scores (*r* = 0.715, *p* < 0.001). To investigate the relationship between lip desquamation and age, we further analyzed the facial images of 1,000 individuals. A gradual decline in lip desquamation with age was observed in males (*r* = -0.151, *p* < 0.001) and females over 38 years (*r* = -0.133, *p* < 0.05). In contrast, the variation of lip desquamation among females under the age of 38 showed an increasing trend (*r* = 0.234, *p* < 0.01). It is notable that the most pronounced differences between sexes were only observed in individuals in their 20s (*p* < 0.001). Such results imply that the characteristics of epithelial layer of lip can be segmented based on chronological age and sex. These notable changes in lip desquamation were discovered for the first time in this study. Given the current ease of acquiring image data, our image-based method has the potential to advance the lip skin research.

**Supplementary Information:**

The online version contains supplementary material available at 10.1038/s41598-025-89264-x.

## Introduction

The lips are a central feature in the lower region of the facial skin and play a crucial role in perception of the facial appearance^1–4^. Several studies have defined healthy lips by assessing features such as shine, texture, fissures, and redness^5–7^. A recent study revealed that dermatological professionals consider the well-hydrated and smooth lip as the most critical visual indicators of healthy lips^8^. These findings highlight the importance of lip hydration in overall lip health and the need for a comprehensive understanding of lip characteristics. The lip vermilion, distinct from surrounding skin, exhibits unique structural and physiological properties^9^. It serves as transitional area of the oral mucosa that is constantly exposed to the external environment^10,11^. Due to incomplete corneocyte formation, lip vermilion also contains much larger corneocytes compared to other body sites^12,13^, constituting a thin epithelial layer. These features contribute to the weakened barrier function and water-holding capacity of the lip stratum corneum (SC)^14^. Lip SC displays the highest transepidermal water loss (TEWL) on the face, approximately three times higher than that of the facial skin^12,15^. Additionally, the lower lip tends to be drier than the upper lip^16^, and it becomes more hydrated as it reaches closer to the mucosa^17^. The poor functionality of vermillion zone often leads to lip roughness and chapping, accompanied by cracking or bleeding on the lip surface. Such common problems have a significant impact not only on the facial appearance but also on the dermatological health of the lips.

To investigate the distinctive characteristics of lip SC, previous studies have utilized commonly used methods for evaluating other body sites. The first approach involves visually grading the severity of lip dryness using photonumeric scales^18–21^. However, concerns have been raised about the reliability and reproducibility due to its subjective nature^22^. The second strategy is to employ skin-contact devices such as the Tewameter^®^ and the Corneometer^®^ (Courage & Khazaka, Cologne, Germany), to assess the functionality of lip surface^23^. Although they have been considered as objective alternatives for measuring TEWL and water content, the measurements should be conducted under strictly controlled conditions to minimize the influence of external factors^23–25^. Another basic method involves analyzing the image of corneocytes obtained from tape-stripping using the Surface Evaluation of Living Skin (SELS) method^26^. However, the application to the delicate skin of the lips is limited due to the potential damage or discomfort caused by repetitive stripping.

Image processing algorithms have been developed to address limitations of existing methods. Several studies have utilized these strategies to investigate the variability in lip skin and morphology. For instance, Hamer M.A. et al. evaluated the upper lip wrinkles of 150 facial images using image smoothing, thresholding, and line connection algorithms^27^. In another research, three-dimensional facial landmarks were detected to calculate various parameters related to lip vermillion size, area, and volume^28^. In our previous study, we also reported the lip aging features by analyzing the facial images of 1,000 Korean women, using facial landmark detection and pattern recognition algorithms^29^. Although these studies used image analysis techniques to analyze diverse lip properties, there has been limited focus on lip desquamation to date.

This study aimed to develop a quantitative method for assessing lip desquamation using image analysis. Initially, we obtained facial images and collected lip SC flakes through tape-stripping from a total of 55 individuals. By utilizing facial landmarks detection and global thresholding, the desquamated flakes were extracted within the specific lip vermilion region in each facial image. Compared to tape-stripping, the image-based approach showed a significantly stronger correlation with visual assessment in identifying lip surface flakes. Furthermore, we conducted an additional analysis using an extensive dataset of 1,000 facial images from Korean participants to explore the variations in lip desquamation related to age and sex.

## Methods

### Study participants

The first part of the study proceeded with 55 participants, aged 25 to 43 years (mean age 31.3), to acquire facial images and perform tape-stripping. Based on previous studies demonstrating a decrease in lip dryness with age^12^, Korean participants in age 20s to 40s were recruited. Through a preliminary survey, only healthy individuals with no prior history of exfoliative cheilitis were selected as study participants. Accordingly, 34 females and 23 males were initially recruited, but two women were withdrawn due to discomfort during the tape-stripping process. All participants were instructed to refrain from applying any topical lip-care products for 12 h, as well as eating or drinking for one hour before the measurements. Measurements were conducted under controlled conditions, with a constant temperature of 22 ± 2 °C and humidity of 50 ± 10%, in October to November 2023. Participants were asked to gently wipe their lips with a paper towel to remove any impurities and then relax for 10 min to adjust to the condition in the room. For the second part of the study, the facial images of 1,000 individuals, consisting of 500 men and 500 women, were included, all of whom were recruited in our previous study^30^. Consequently, a total of 1,055 individuals were involved in this study.

### Facial image acquisition

Facial images of the 1,055 individuals were taken under identical conditions (Supplementary Fig. 1). Images were taken using a high-resolution camera (Canon 200D DSLR, Tokyo, Japan) of the Janus-III measurement system (PIE, Suwon, South Korea) under normal light conditions. Subjects were photographed in a blackout environment to block any external light sources, with their forehead and chin fixed. During photography, subjects were instructed to wear a light-blocking drape to control potential influencing factors. For the facial images of 1,000 individuals from our previous study^30^, we randomly selected 10 men and 10 women from each age group, ranging from 20 to 69 years old.

### Tape-stripping of lip surface

After taking facial images, desquamated flakes were sampled from each participant by tape-stripping the surface of lower lip center below mucosa. The Black D-squame^®^ rectangle tape (CuDerm, Dallas, TX, USA) was used for stripping. The corneocytes collected on the tape were assessed using Visioscan^®^ VC 98 (Courage & Khazaka, Cologne, Germany) and its analysis software, which visualizes skin roughness based on the thickness of corneocytes by exploiting their differential absorption of ultraviolet light. The software automatically classifies the corneocytes into five groups from G0 (even layer) to G4 (thick flakes), ranging from grayscale values of 80 (default) to 255 in 35-unit intervals, and calculates the proportion of each group within the total area. To obtain optimized data, it is allowed for users to manually adjust the cutoff value for each group^31^. As a quality control process for data reliability, we excluded corneocytes with grayscale values lower than 150, specifically those in the G0 (grayscale value 80–115) and G1 (115–150) groups. Consequently, the desquamation rate determined by the tape-stripping method was defined as the total proportion of detected flakes in the G2, G3, and G4 groups for each participant.

### Visual assessment (VA) of lip desquamation

**Fig. 1:**
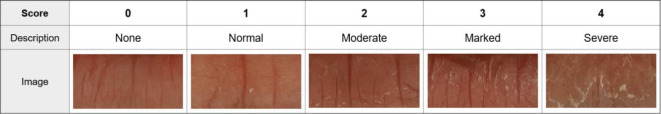
Criteria for the visual assessment of lip desquamation. Each score indicates the degrees of desquamation in the target lip region, with corresponding descriptions and representative images provided above. The clinical implications of each score, ranging from 0 to 4, are outlined as follows: 0: None (no chapping or desquamated flakes); 1: Normal (very few small flakes); 2: Moderate (a few small flakes); 3: Marked (obvious flakes in various sizes and shapes); 4: Severe (very marked scaling, mainly forming large flakes).

Lip images from 55 participants showed a clear progression of desquamation. According to this observation, we established a scoring criteria, ranging from a score of 0 to 4, where each score represents five distinct levels of desquamation (Fig. 1). Based on the criteria, five dermatology researchers independently evaluated the degree of lip desquamation for 55 participants. The correlation coefficients among the score sets were all above 0.75 (*p* < 0.01) with an average coefficient of 0.827, indicating a high inter-rater reliability (Supplementary Fig. 2). The final VA score for every participant was defined as the average value of the five scores, resulting in a predominantly ranged scores between 1 and 3 points (Supplementary Table 1).

### Data analysis

**Fig. 2:**
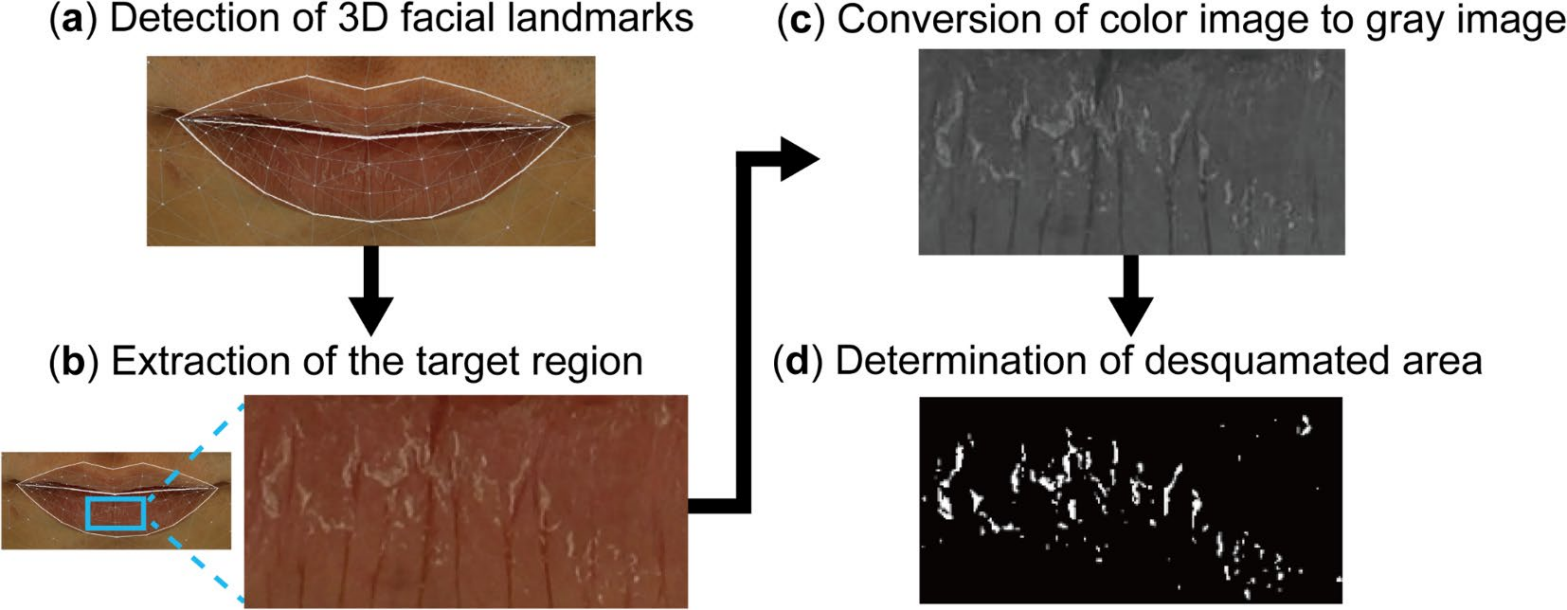
Schematic workflow of the image-based method for quantifying the lip desquamation. (**a**) The facial landmarks were detected. (**b**) The target lip region was cropped from facial image using the detected landmarks. (**c**) The color space of the image is then converted from RGB to grayscale. (**d**) By applying global thresholding, desquamated flakes are segmented out as white pixels in the binary image. The desquamation rate is calculated as the percentage of white pixels among all the black and white pixels.

All the statistical analyses, including tests for normality, correlation, and regression, were performed using R Statistical Software (v.4.0.3; R Core Team 2020)^32^. The normality of the data was assessed using the Shapiro-Wilk test. Pearson’s correlation test was used to compare the performance among the three methods: VA, image analysis and tape-stripping. In the analysis of the images of 1,000 individuals, we utilized local regression analysis to visualize the results and highlight the age and sex-related changes more specifically. Spearman’s non-parametric correlation test and linear regression test were also used for further analysis. To compare the desquamation level among different age groups, Kruskal-Wallis test and Wilcoxon rank sum test were used. Statistical significance level of *p* < 0.05 was used for all analyses. All the figures, except Figs. 1 and 2, were visualized using ggplot2 package^33^.

### Image analysis

To automatically detect the lip area from facial image, we used Google MediaPipe, an open-source framework for building machine learning pipelines that focuses on various computer vision applications^34^. Among the various models available in MediaPipe, we used the Face mesh model from the Face Landmarker in Python 3.7.5 to predict 3D facial landmarks. This model provides an estimate of 478 facial landmarks with corresponding coordinates. We selected eight landmarks numbered 14, 15, 16, 17, 84, 87, 314 and 317, which together represent the center area of the lower lip (Supplementary Fig. 3). The target region for analysis was extracted in a rectangular shape. We calculated the x-coordinate spans between landmarks 84 and 87, and between landmarks 314 and 317, defining the larger value as the width of the target region. The height of the target region was calculated as the y-coordinate difference between the midpoints of landmarks 14–15, and 16–17. After cropping the target region from original image, all the subsequent image processing analyses were conducted using the Imager R package (v.0.42.13)^35^, including RGB-to-grayscale conversion and global thresholding.

### Ethics declarations

The study was conducted in accordance with the Declaration of Helsinki, and approved by the Institutional Review Board (IRB) of LG H&H Research Center, Seoul, South Korea (LGHH-20231019-AB-03-01). All methods were performed in accordance with the relevant guidelines and regulations. Informed consent was obtained from all participants involved in the study, including their permission for the publication of information and images in an online open access publication.

## Results

### Development of quantification method based on image analysis

#### Workflow of the image-based method

To specify the lip area from facial images, we applied Google MediaPipe^34^ to detect 3D facial landmarks (Fig. 2a). Among the 478 landmarks detected, eight landmarks corresponding to the center of the lower lip were utilized to extract the target lip region (Fig. 2b, Supplementary Fig. 3, Methods). Subsequently, the target region was cropped from the facial image and was converted from RGB to grayscale (Fig. 2c), to manifest the differences between normal and desquamated areas. In the grayscale images, exfoliated flakes appeared brighter compared to the adjacent lip skin areas. Therefore, we applied global image thresholding^36^ to segment the flakes based on their brightness. Specifically, pixels with gray levels below and above the threshold ($\:\text{T}$) were assigned to the background (black pixels, gray level 0) and the objects (white pixels, gray level 255), respectively. The final image contained only black and white pixels, each representing the normal and desquamated areas on the lip surface (Fig. 2d). The desquamation rate was calculated as the percentage of white pixels in the binary image, which provides a quantitative measure of lip desquamation in the target lip region (Supplementary Fig. 4).

#### Determination of the optimal threshold value

**Fig. 3:**
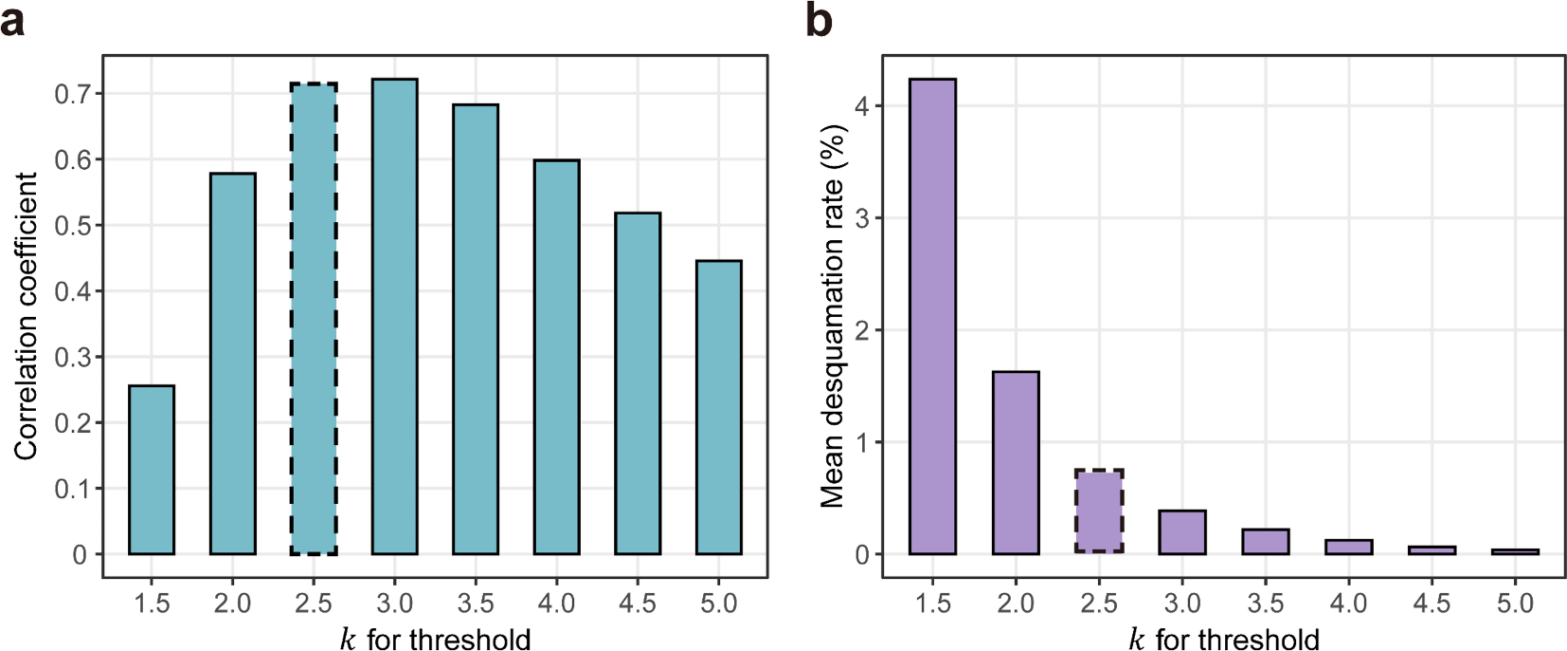
Performance of the image-based method using different threshold values ($\:\text{T}$). (**a**) The correlation coefficients (y-axis) were calculated to assess the relationship between the desquamation rates and the VA scores. Desquamation rates were obtained using different threshold values ($\:\text{T}$), which were derived from the different values of parameter $\:k$ (x-axis). (**b**) Each purple bar represents the mean desquamation rate calculated using different values of parameter $\:k$. This indicates that increasing $\:k$ values for thresholding decreases the detection of desquamated areas, potentially leading to the omission of existing flakes on the lip surface. The correlation coefficient and mean desquamation rate derived from the optimal parameter value $\:k$ = 2.5 are denoted by dashed outlines in both (**a**) and (**b**).

In the images of target lip region, we found the flakes resemble the skin defects such as pigment spots or wrinkles. For example, the flakes constituted only a tiny portion in each lip image, with varying shapes and gray levels. Additionally, grayscale intensity of flakes followed a long-tailed unimodal distribution rather than a bimodal distribution (Supplementary Fig. 4). These outlier-like features made it challenging for automatic thresholding algorithms to accurately extract the desquamated flakes^37,38^. In our analysis, conventional algorithms such as Otsu’s^39^ and Triangle^40^ thresholding mistakenly detected the normal regions as desquamated area (Supplementary Fig. 5). Therefore, to find the threshold values ($\:\text{T}$) for the different lip images, we utilized an outlier detection method based on standard deviation ($\:\text{S}\text{D}$). The equation for $\:\text{T}$ was derived as follows:

$$\:\text{T}\:=\:\text{M}\text{e}\text{a}\text{n}+k\text{*}\text{S}\text{D},$$

where $\:\text{M}\text{e}\text{a}\text{n}$ and $\:\text{S}\text{D}$ are the average and standard deviation of grayscale values of each pixel, respectively. The constant variable $\:k$, is a control parameter ranging from 1.5 to 4.5 with increments of 0.1. Using the values of $\:\text{T}$ derived from the equation, we calculated the desquamation rates and performed correlation analysis with visual assessment (VA) scores (Fig. 3a, Supplementary Fig. 6). The correlation coefficient rapidly increased for $\:k$ values ranging from 1.5 to 2.3, and consistently remained above 0.7 for $\:k$ within the range of 2.4 to 3.4. Subsequently, a gradual decrease in correlation coefficient was observed over $\:k$ = 3.1. In contrast, as threshold values increased, the mean desquamation rate, which represents the total detected desquamated areas, decreased (Fig. 3b). Therefore, to maximize the correlation with VA scores and minimize the potential omission of existing SC flakes, we determined $\:k$ = 2.5 to be the optimal parameter for threshold. Consequently, the desquamation rates and VA scores showed a significantly strong correlation (*r* = 0.715, *p* < 0.001).

#### Performance comparison with tape-stripping

**Fig. 4:**
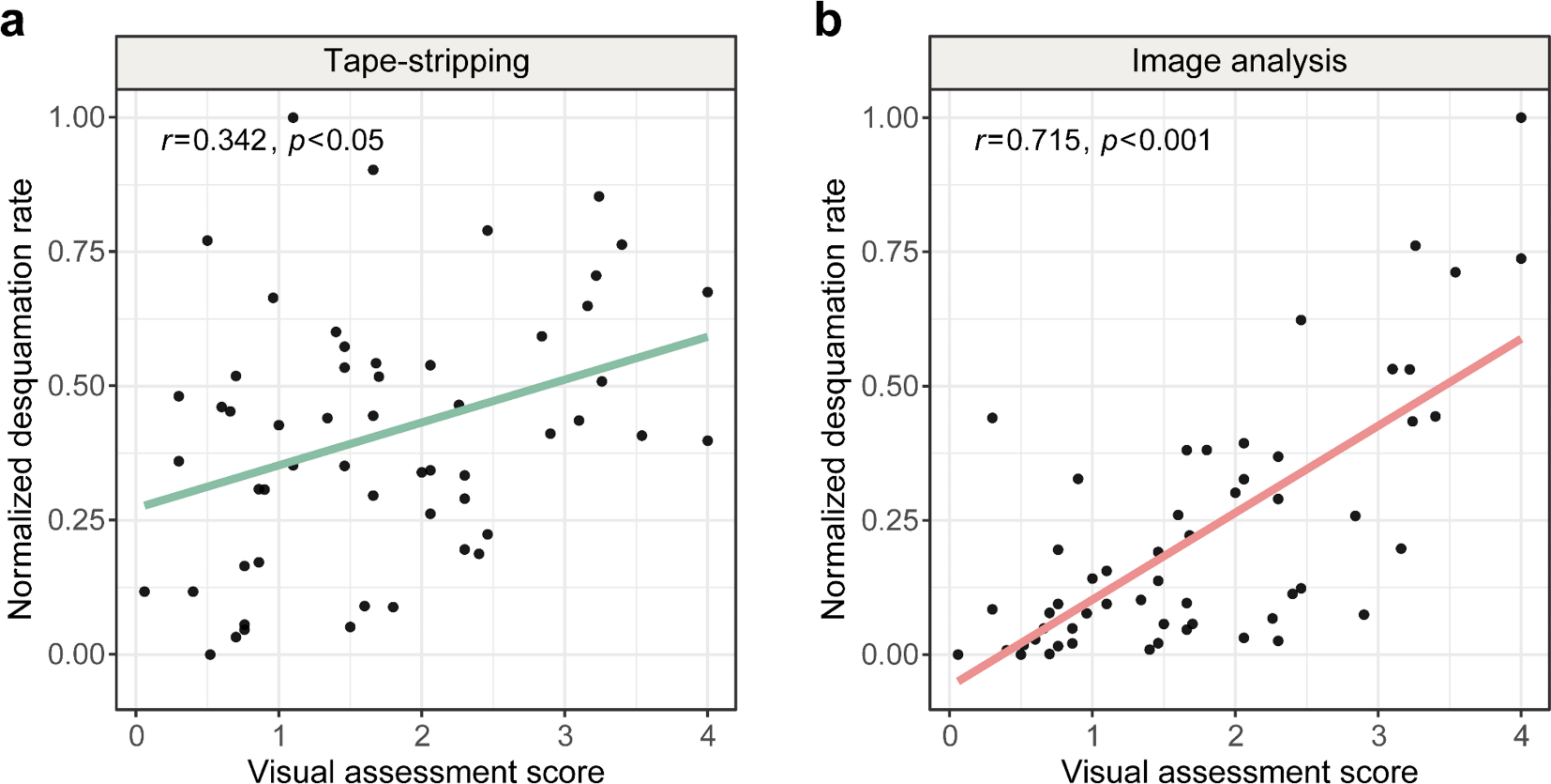
Correlation between VA scores and the desquamation rates derived from the image analysis and tape-stripping. The normalized desquamation rates derived from the tape-stripping method (**a**) and the image-based method (**b**) are plotted on the y-axis. The correlation coefficient *r* and *p*-value of (**b**) (*r* = 0.715, *p* < 0.001) are identical to those of Fig. 3a corresponding to $\:k$ = 2.5, except for the normalization of the desquamation rate. The VA scores of the participants are plotted on the x-axis. The green and red solid lines represent the linear regression lines fitted to the corresponding data, with *r* and *p* indicating the correlation coefficient and p-value, respectively.

Tape-stripping is one of the traditional methods for quantifying desquamation. Using the VA score as the ground truth, we compared the image-based method with the tape-stripping method. A correlation analysis was conducted between the desquamation rate obtained using the tape-stripping method and the VA score (Fig. 4a). Although the results demonstrated a positive relationship, a relatively weak correlation was observed (*r* = 0.342, *p* < 0.05). In contrast, image analysis exhibited a much stronger and statistically significant correlation with the VA score, which is about twice as high as that of tape-stripping method (*r* = 0.715, *p* < 0.001) (Fig. 4b). In addition, we compared the mean squared error (MSE) of the two methods, which measures the average deviation of each measured value from the regression line, indicating how well the model fits the observed data. The measured desquamation rate values of image analysis exhibited more concentrated distribution around the regression line (MSE = 0.026) than that of tape-stripping (MSE = 0.049). These results suggest that the image-based method is a more accurate and robust approach for detecting and quantifying the exfoliated flakes on the lip surface, compared to the traditional tape-stripping method.

### Analysis of lip desquamation according to age and sex

**Fig. 5:**
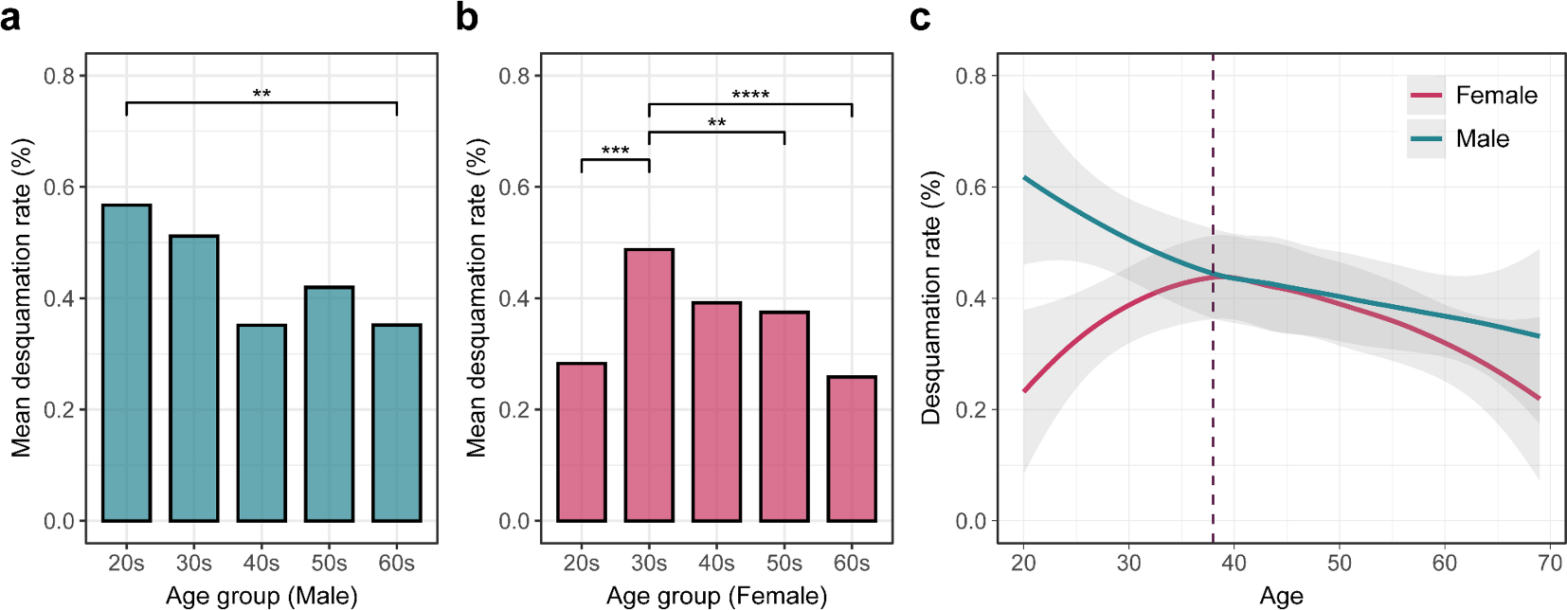
Lip desquamation level according to age and sex among 1,000 individuals. (**a**), (**b**) The mean desquamation rates for different age groups in males and females, respectively. Significant differences in pairwise comparison are denoted by asterisks (*p-value < 0.05, **p-value < 0.01, ***p-value < 0.001, and ****p-value < 0.0001). (**c**) The solid lines represent the nonlinear regression lines for males (blue line) and females (red line), with the gray shaded area indicating the 95% confidence interval. The vertical dashed line indicates the age of 38, where females start to show a decline in desquamation rate.

To investigate age and sex-related changes in lip desquamation, we applied our image-based method to facial images of 1,000 individuals. Overall, males exhibited higher mean desquamation rates than females, with 0.440% and 0.359%, respectively (Fig. 5a, b). Specifically, males and females in their 20s showed a statistically significant difference in desquamation rates (*p* < 0.001, Supplementary Table 2). This indicates that the young age group is the primary contributor to the overall difference in lip desquamation between sexes. Furthermore, the age-related variation of lip desquamation showed different trends between males and females (Fig. 5c). Males exhibited a negative correlation between the desquamation rate and age (*r* = -0.151, *p* < 0.001, Supplementary Fig. 7a), with a significant difference observed between the 20s and 60s age groups (*p* < 0.01, Fig. 5a, Supplementary Fig. 7b). In contrast, females showed an upward convex trend, reaching the maximum value in late 30s (Fig. 5c, Supplementary Fig. 8). To specify the inflection point in females, we calculated the p-values of differences in slopes between the linear regression lines for female groups before and after each age. Notably, the highest level of significance was observed at age 38, suggesting that the slopes of linear regression lines for females under and above 38 years showed the largest difference (Supplementary Fig. 8). Specifically, females exhibited an increasing pattern until the age of 38 (*r* = 0.234, *p* < 0.01), followed by a gradual decline thereafter (*r*=-0.133, *p* < 0.05, Supplementary Fig. 7c). In addition, the desquamation level in the 30s was significantly higher than in the 20s, 50s and 60s for females (*p* < 0.01, Fig. 5b, Supplementary Fig. 7d).

We also conducted linear regression analyses for each sex, as shown in Supplementary Fig. 9. While males showed a decline with age, the two groups of females exhibited a significant difference in their slopes, with values of 0.019 and − 0.005, respectively. The decreasing trend after the age of 38 was highly consistent between males and females, with similar estimated slopes of -0.005. Furthermore, the estimated desquamation rate for females aged 38 (0.552%) was approximately 2.5 times higher than that for females aged 20 (0.217%). Thus, lip desquamation in adults can be categorized into two main patterns, the increasing desquamation of females in 20s to 30s, and the decrease in males and other females.

## Discussion

Lip is one of the central features in the face, and greatly contribute to the facial appearance^1–4^. Recently, several studies, including our previous study, have utilized image processing algorithms to explore the morphological aspects of lip^27–29^. However, there has been a lack of focus on desquamation of lip surface. Leveraging the large-scale image data and analysis algorithms, we proposed a quantitative image-based method for measuring the lip desquamation in this study. The facial images of 55 individuals were analyzed using the method, and we assessed the performance compared to the tape-stripping method. Furthermore, we applied our approach to the facial images of 1,000 individuals, to investigate the age and sex-related changes of lip desquamation.

We focused on the driest region of the lip, where we expected a higher severity of lip desquamation. According to the previous studies that the lower lip is drier than the upper lip, we selected the lower lip vermilion zone as the primary area for analysis^16^. Specifically, we determined a predefined area on the lower lip, known as the premucosa, as the target region for our study. Caisey et al. demonstrated that the premucosa, located closest to the oral mucosa in the lower lip, is less hydrated than the outer lip skin^17^. To mitigate the variations in lip morphology, we used 3D facial landmarks for precise identification of the target lip region in each image. Using the specific lip landmarks, the target lip region was extracted in a rectangular shape that matched the tape-stripped area.

In skin sites, scaly areas generally appear brighter compared to the smooth areas, which has led to the development of skin scaliness indices^41,42^. Similarly, in the lip images, we found that the flakes on the lip surface also exhibited a higher level of brightness than the surrounding lip area. Therefore, we utilized global thresholding strategy to separate the image into the desquamated flakes (objects) and the normal lip area (background), based on their grayscale distribution^36^. To determine the optimized threshold values ($\:\text{T}$) for each lip image, we utilized $\:\text{S}\text{D}$-based method instead of using a fixed value for all images^43^. During the performance test for the parameter $\:k$, the values of 2, 2.5, and 3 were determined as the initial candidates due to their computational convenience^44,45^. The desquamation rates derived from both 2.5 and 3 exhibited significant correlation coefficients above 0.7 with VA scores. However, as the value of $\:\text{T}$ increases, the total desquamated areas detected from our method decreased. Therefore, we selected the smaller value, $\:k$ = 2.5, as the optimal threshold parameter to minimize the loss of detection.

Compared to tape-stripping, the image-based method showed a stronger correlation with VA. This suggests that our method more accurately represents the condition of the lip surface than tape-stripping method. It also indicates that tape-stripping has limitations for quantifying the exfoliated lip, which can be supported by previous studies. Firstly, the tape-stripping method analyzes the corneocytes removed from the skin site^26,31^, which may alter the original state of the flakes during the stripping process. Both intrinsic and extrinsic factors such as the lipid composition of skin and stripping pressure can also influence the results^41,42^. Given that lip skin has numerous wrinkles and is susceptible to chapping or cracking, the extraction of distinct cell layers and interpretation of the results can also be hindered^12,46,47^. Furthermore, tape-stripping can irritate the skin depending on the surface condition^48^. In our experiments, two participants eventually discontinued the measurement due to pain. This implies that tape-stripping is partially invasive for delicate skin areas including lip surface, although it is considered as a non-scarring method for other normal skin regions^49^. Therefore, considering the accuracy and non-irritating nature, our method can serve as an alternative approach for quantifying lip desquamation.

The analysis of 1,000 facial images revealed differences between males and females in the age-related changes of lip desquamation. Among males, the desquamation rates gradually decreased with age, showing a significant difference between 20s and 60s. This result is partially supported by the association between lip dryness and transepidermal water loss (TEWL), which has been reported to decrease with age^12,50^. Notably, this study is the first to identify the specific tendency of lip desquamation in males, as previous research has mainly focused on females. A more comprehensive investigation of male lip is required.

Lip desquamation in females increased until the age of 38 and subsequently decreased. This result partially confirms the previously reported trends for female lips. Tamura et al. revealed that the lip dryness problem is most prevalent among females in 30s, with significantly higher levels than 40s, following the same tendency as TEWL^50,51^. Our result aligns with a previous study that showed higher TEWL values of females in 30s than 50s to 70s^50^. The changes of hormones and keratinocyte ceramide can be significant factors causing such tendencies. Estrogen levels are known to peak in the mid- to late 20s in females, followed by a gradual decline until the onset of menopause^52^. Specifically, oestradiol (E2), a type of estrogen, is closely involved in the synthesis of keratinocyte ceramide (CER), which greatly impacts the epidermal barrier function^53^. According to a previous study, lip roughness is also related to the levels and carbon numbers of specific kinds of CERs^54^. These findings can be associated with the distinct trend of lip desquamation among young groups of females, which contrasts with the gradual decrease shown in males.

In summary, we developed a method for quantifying the lip desquamation from facial images and evaluated the performance. We found that our method preserves the visible state of lip surface more reliably than tape-stripping method. We further applied the method to the images of 1,000 individuals. Males showed a gradual decrease in lip desquamation with age, which is the first to be identified. In contrast, females exhibited an increasing trend up to the age of 38, followed by a subsequent decrease. The image-based approach enables a convenient, objective and non-irritating quantification of the desquamated lip flakes. It has the potential to be an alternative to tape-stripping and visual assessment for lip skin. Our study will contribute to a better understanding of lip desquamation and the development of relevant treatments for lip in both fields of dermatology and cosmetics.

## Electronic supplementary material

Below is the link to the electronic supplementary material.

Supplementary Material 1

## Data Availability

The datasets generated during the current study are not publicly available due to privacy concerns related to human facial data, but are available from the corresponding author on reasonable request.
