# Supplementary material for: Image based quantification method reveals differential patterns of lip desquamation associated with age and sex
Source: Sci Rep. 2025 Apr 8;15:11927. doi: 10.1038/s41598-025-89264-x (PMC11977245; doi:10.1038/s41598-025-89264-x)
Supplement: Supplementary file 1 — Supplementary Material 1 [file 41598_2025_89264_MOESM1_ESM.pdf]

Supplementary Information

Image based quantification method reveals differential patterns of lip desquamation associated with age and sex

Hanji Kim[1], Jung Yeon Seo[1], Sangseob Leem[1], Seung Won You[1], Yunkwan Kim[1,*] and Nae Gyu Kang[1,*]

[1] Research and Innovation Center, R&D Institute, LG Household & Health Care (LG H&H), Ltd, Seoul, 07795, Republic of Korea

*Correspondence: kimyoonkwan@lghnh.com (Y.K.), ngkang@lghnh.com (N.G.K.)

Contents:

Supplementary Figures. 1-9

Supplementary Tables. 1-2

Supplementary Figures

Supplementary Fig. 1. Facial image acquisition under the Janus-Ⅲ measurement system

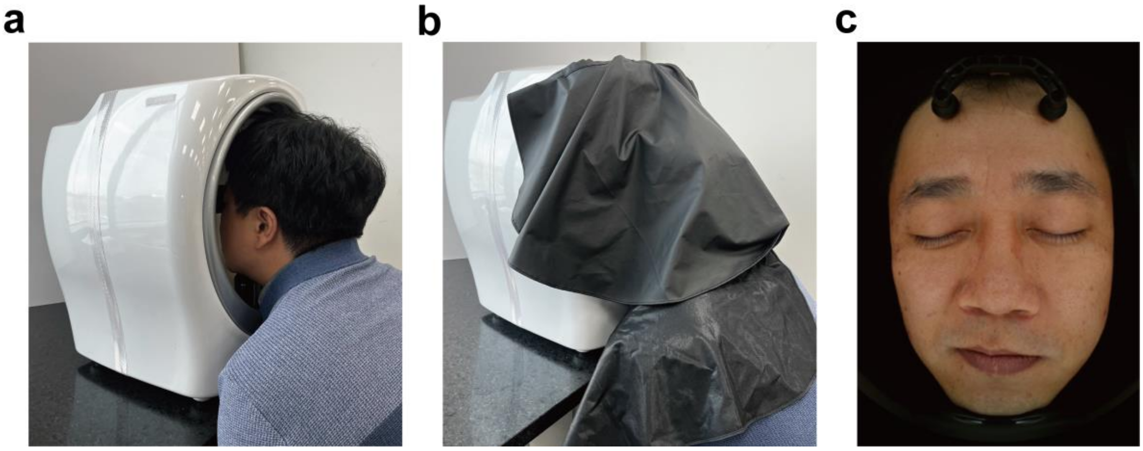

a. Facial images were generated using the Janus-Ⅲ measurement system (PIE, Suwon, South Korea) under controlled conditions. In this system, subjects are photographed with their forehead and chin fixed in the device, keeping their face in a stationary position. **b.** During photography, subjects were instructed to wear a blackout drape to prevent potential intervening factors. **c.** As a result, a facial image is provided with all external light sources blocked, except for the built-in normal light source.

Supplementary Fig. 2. The correlation coefficients among the visual assessment (VA) score sets provided by the five assessors.

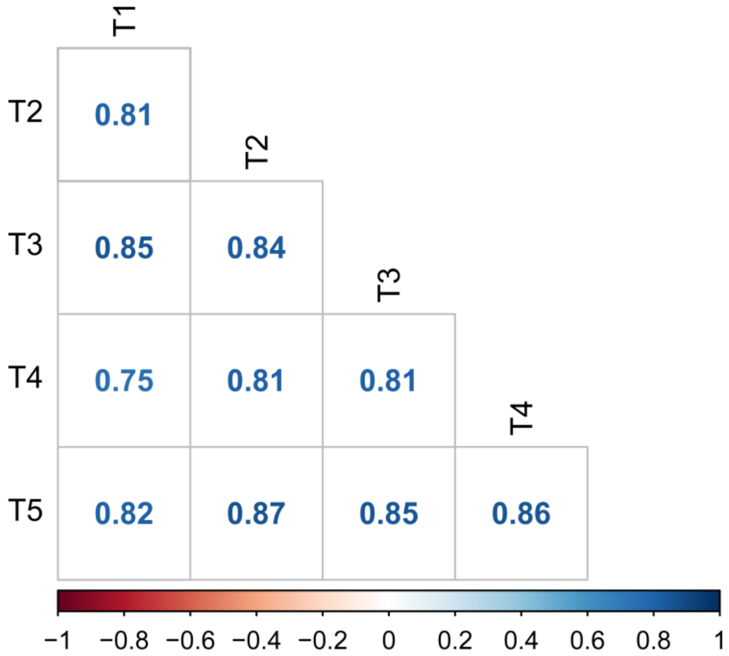

A statistically significant correlation was observed among the VA scores provided by the five assessors ($r \geq 0.75$, $p < 0.001$). The average correlation coefficient was 0.827, indicating a high level of inter-rater reliability among the assessors in VA of 55 participants.

Supplementary Fig. 3. Landmarks used for detecting the target lip region

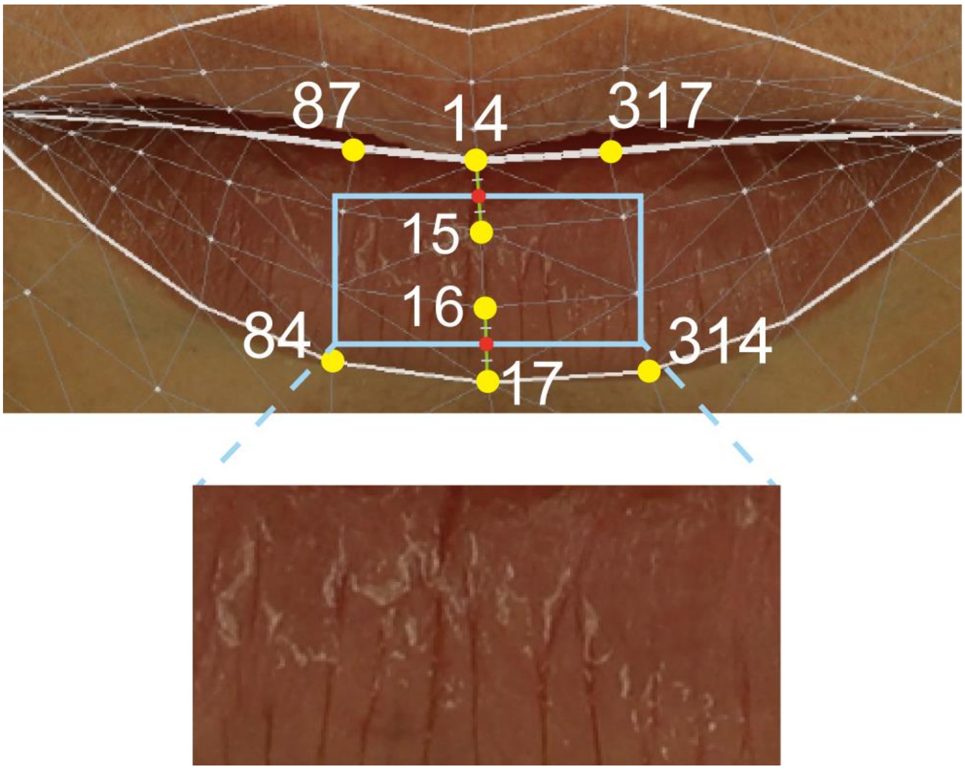

The 478 facial landmarks predicted from MediaPipe are shown, with the 8 landmarks (in yellow and green) representing the central region of the lower lip. The green landmarks are numbered 15 and 16, respectively, and are located in sequence with landmarks 14 and 17. The rectangular target lip region is defined using the coordinates of these landmarks. The width of the target region is determined by the maximum x-coordinates between the landmarks 84 and 87, and between landmarks 314 and 317. The height is determined by the distance between the midpoint of landmarks 14 and 15, and the midpoint of landmarks 16 and 17.

Supplementary Fig. 4. Quantification of the lip desquamation in target lip region image.

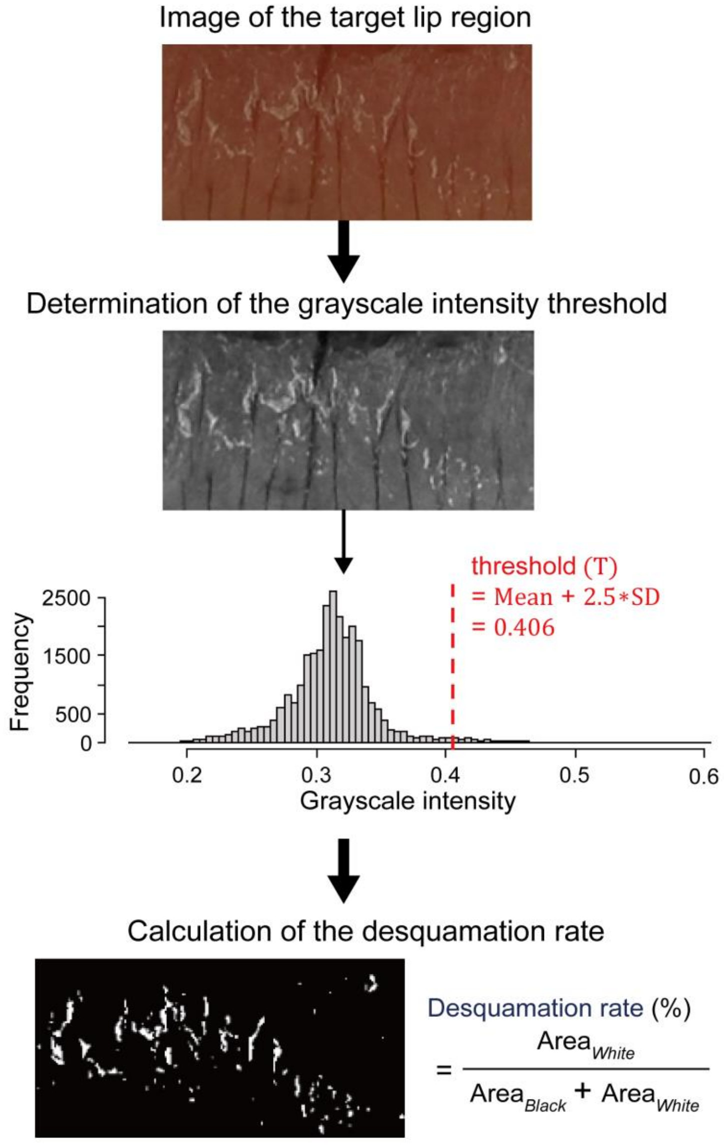

The target lip region is extracted from the original facial image and converted into grayscale image. The threshold value (T) is determined as $\mathrm{Mean} + 2.5 * \mathrm{SD}$ of grayscale intensity values for all pixels (see the grayscale intensity histogram), where Mean and SD are the average and standard deviation of grayscale values of each pixel, respectively. Global thresholding is performed based on predefined T value, resulting in a binary image of the target lip region. Finally, the desquamation rate is calculated as the percentage of white pixels to the total number of pixels in the image.

Supplementary Fig. 5. Binary images derived from three different thresholding strategies.

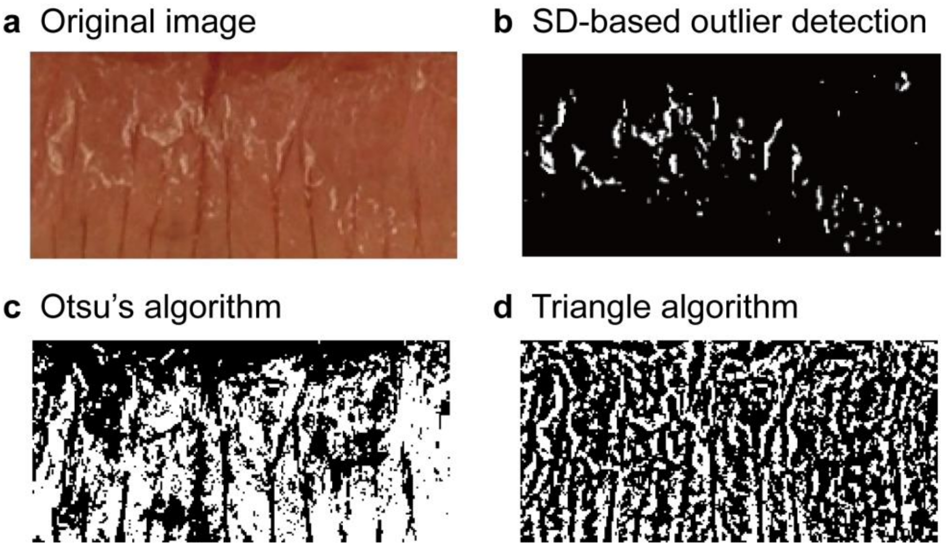

a. The original image of the target lip region. **b.** The binary image derived from global thresholding based on standard deviation (SD)-based outlier detection. We utilized this method for our analysis. **c,d.** The binary images obtained using two automatic thresholding algorithms, Otsu’s algorithm (c) and the Triangle algorithm (d). The application of these algorithms was performed using the *auto_thresh()* function in autothresholdr, with default parameters [1].

Supplementary Fig. 6. Performance of the image-based method with parameter k.

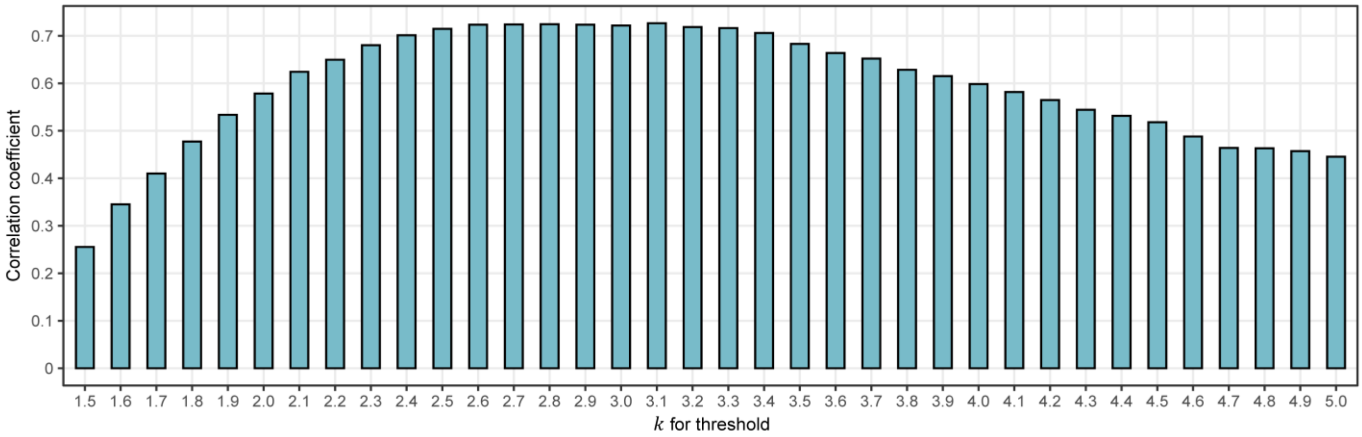

The height of each bar indicates the correlation coefficient (y-axis) between the VA score and the desquamation rate. The desquamation rate was measured using the threshold (T), which is derived from the corresponding value of parameter k (x-axis), ranging from 1.5 to 5.0.

Supplementary Fig. 7. Relationships between age and lip desquamation rates of 1,000 individuals.

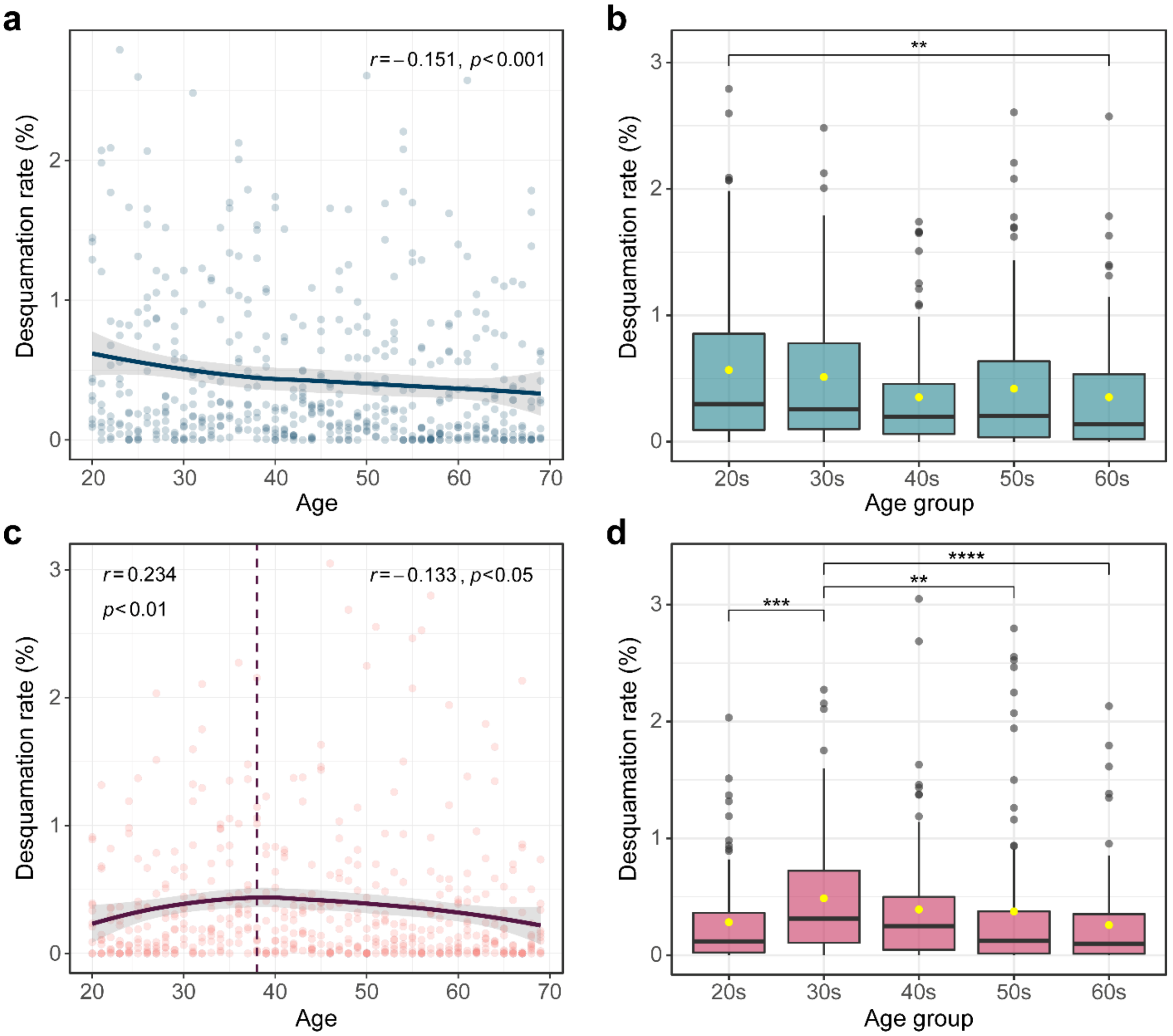

a,c. The tendencies of lip desquamation according to age are described for 500 males (a) and 500 females (c). The solid line represents the nonlinear regression that fits the data, with the gray shaded area indicating the 95% confidence interval. The values of *r* and *p* represent the correlation coefficient and p-value, respectively. **b,d.** Boxplots display the desquamation level for different age groups in males (b) and females (d). The average value of each group is marked as yellow points. Significant differences in pairwise comparison are denoted by asterisks (*p-value <0.05, **p-value < 0.01, ***p-value < 0.001, and ****p-value < 0.0001).

Supplementary Fig. 8. Statistical significance of slope difference between two regression lines before and after a specific age.

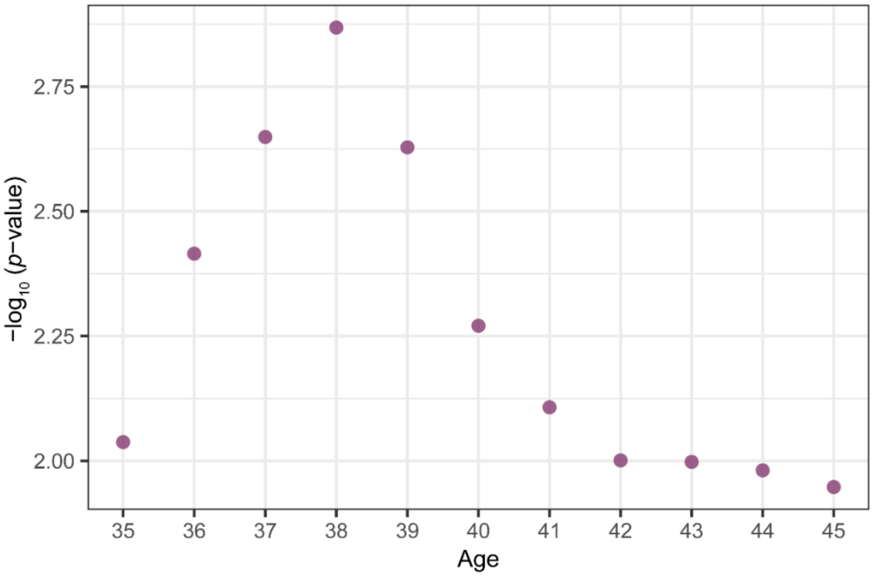

The p-values of the differences in slope between the linear regression lines were calculated for the groups before and after each age (x-axis), ranging from 35 to 45. The obtained p-values were transformed into their negative logarithmic form and plotted on the y-axis. The highest value was observed at the age of 38, indicating the most significant difference in slopes before and after that age.

Supplementary Fig. 9. Linear regression analyses of males and females.

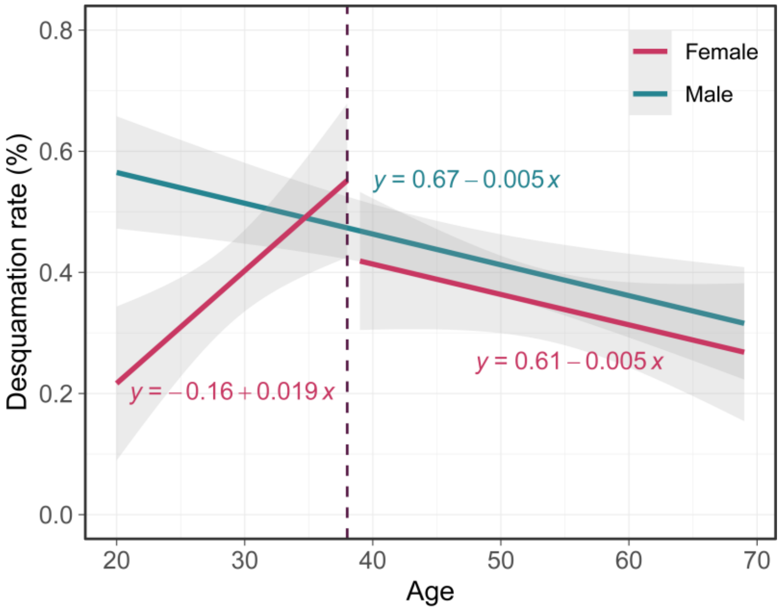

The solid lines represent the linear regression lines that fit the desquamation rates of male group and two female groups, with the gray shaded area indicating the 95% confidence interval. The desquamation rate of males (blue line) gradually decreased, while females (red lines) showed an increase until the age of 38, followed by a decreasing trend. After the age of 38, the desquamation rate of both genders exhibited a similar tendency to decrease, with a similar slope value of -0.005.

Supplementary Tables

Supplementary Table 1. A statistical summary of the visual assessment (VA) scores for all participants.

Score (Grade)	0	1	2	3	4
Description	None	Normal	Moderate	Marked	Severe
Female (%)	2 (6%)	12 (38%)	13 (41%)	2 (6%)	3 (9%)
Male (%)	3 (13%)	8 (35%)	6 (26%)	6 (26%)	0 (0%)
Mean age (*SD*)	32.6 (5.3)	30.8 (4.2)	30.9 (3.1)	33.4 (4.8)	30.7 (3.1)

SD, standard deviation

Supplementary Table 2. A statistical summary of lip desquamation rates among 1,000 individuals for different age groups.

**Age group**	**Mean (*SD*)**		***p-value***
	Female	Male	
20s (20–29)	0.283 (0.382)	0.567 (0.640)	<0.001
30s (30–39)	0.487 (0.522)	0.512 (0.559)	n.s.
40s (40–49)	0.392 (0.521)	0.352 (0.425)	n.s.
50s (50–59)	0.375 (0.627)	0.419 (0.552)	n.s.
60s (60–69)	0.258 (0.399)	0.352 (0.470)	n.s.

SD, standard deviation of each age group.

p-value, the significance of the Wilcoxon rank sum test between males and females in the corresponding age group;

Non-significant differences are denoted as n.s.

References

1. Landini, G., Randell, D. a., Fouad, S. & Galton, A. Automatic thresholding from the gradients of region boundaries. *J. Microsc.* **265**, 185–195 (2017).
